# Data in support of covalent attachment of tyrosinase onto cyanuric chloride crosslinked magnetic nanoparticles

**DOI:** 10.1016/j.dib.2016.11.035

**Published:** 2016-11-18

**Authors:** Kourosh Abdollahi, Farshad Yazdani, Reza Panahi

**Affiliations:** Chemistry & Chemical Engineering Research Center of Iran (CCERCI), Tehran, Iran

## Abstract

Preparation and characterization of cross linked amine-functionalized magnetic nanoparticles as an appropriate support for covalent immobilization on tyrosinase was presented in the study "Covalent immobilization of tyrosinase onto cyanuric chloride crosslinked amine-functionalized superparamagnetic nanoparticles: synthesis and characterization of the recyclable nanobiocatalyst" (Abdollahi et al., 2016 ) [1]. Herein, complementary data regarding X-ray powder diffraction (XRD) to characterize the synthesized magnetic nanoparticles, and transmission electron microscopy (TEM) to determine the size and morphology of tyrosinase immobilized magnetic nanoparticles (tyrosinase-MNPs) were reported. The purification results of the extracted tyrosinase from mushroom *Agaricus bisporus* were provided in a purification table. The covalent immobilization of tyrosinase onto cyanuric chloride functionalized magnetic nanoparticles was proved by performing thermo-gravimetric and energy-dispersive X-ray spectroscopy analyses. The operational stability of immobilized tyrosinase was investigated by incubating tyrosinase-MNPs at different pH and temperatures.

**Specifications Table**

**Table:** 

Subject area	Environmental biotechnology
More specific subject area	Enzyme immobilization.
Type of data	Table (purification table), images (TEM, XRD), Figures (TGA, operational stability of immobilized tyrosinase).
How data was acquired	X-ray diffraction of the dried samples with scanning range from 4° to 70° (Bruker D8 Advance, with Cu Kα radiation, *λ*=0.154060 nm), transmission electron microscopy (TEM), operating at 220 KV, vibrating sample magnetometer (VSM, Meghnatis Kavir Kashan Co., Iran), Thermo-gravimetric analysis (TGA) (Netzsch – TGA 209F1 instrument), Scanning Electron Microscope (SEM) equipped with EDX detector (TESCAN Vega Model), UV–vis spectrophotometer (Perkin-Elmer-Lambda 35).
Data format	Analyzed.
Experimental factors	Synthesized magnetic nanoparticles were dried for X-ray diffraction analysis; tyrosinase-MNPs were dried under vacuum at 45 °C and used as a sample for TEM and EDX analyses; TGA analysis was performed on the dried tyrosinase-MNPs; The operational stability of the immobilized tyrosinase was investigated by incubating tyrosinase-MNPs at different pH values and temperatures. The tyrosinase-MNPs were added to a phenolic solution to determine the dephenolization capacity of them.
Experimental features	For stability tests, appropriate amount of immobilized tyrosinase was incubated in different pH values (4.0–8.0) and temperatures (25–65 °C) for 2 h then, the particles were separated and their activities were measured at optimum condition.
Data source location	Chemistry & Chemical Engineering Research Center of Iran (CCERCI), Tehran, Iran.
Data accessibility	Data is represented within this article.

**Value of the data**

• Results show the size and morphology of tyrosinase immobilized nanoparticles, which is important for any application.
• The data of EDX analysis may help to confirm successful immobilization of biomolecules to the surface of nanocarriers.
• Operational stability is playing an integral role in practical application of enzymes in some industrial processes and could be useful as a references and comparisons for other researchers who are working on enzyme immobilization process.
• Data of thermogravimetric analysis (TGA) as well as FT-IR spectra are employed to characterize materials, modified surface and functionalized materials by demonstrating changes in chemical structures.

## 1 Data

**Fig. 1:**
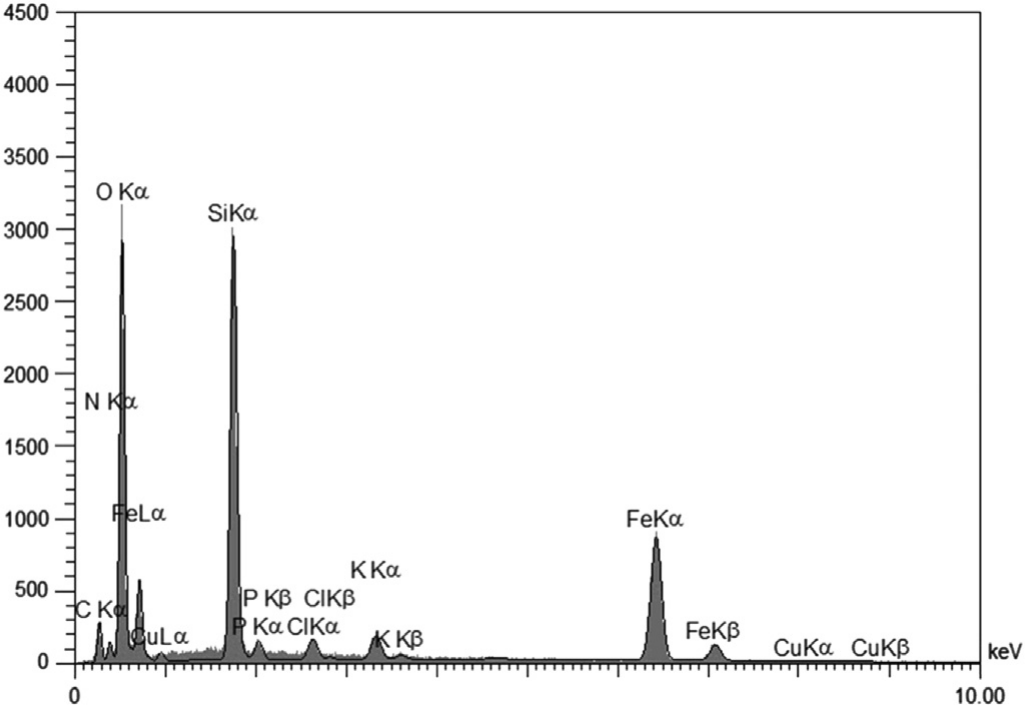
EDX spectrum of immobilized tyrosinase on magnetic nanoparticles.

**Fig. 2:**
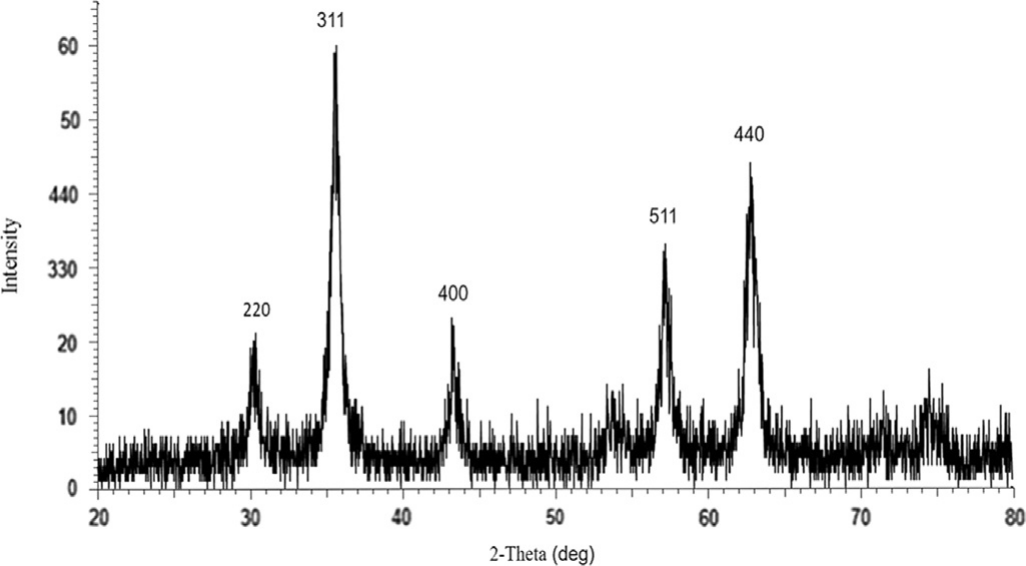
XRD pattern of the bare Fe_3_O_4_.

**Fig. 3:**
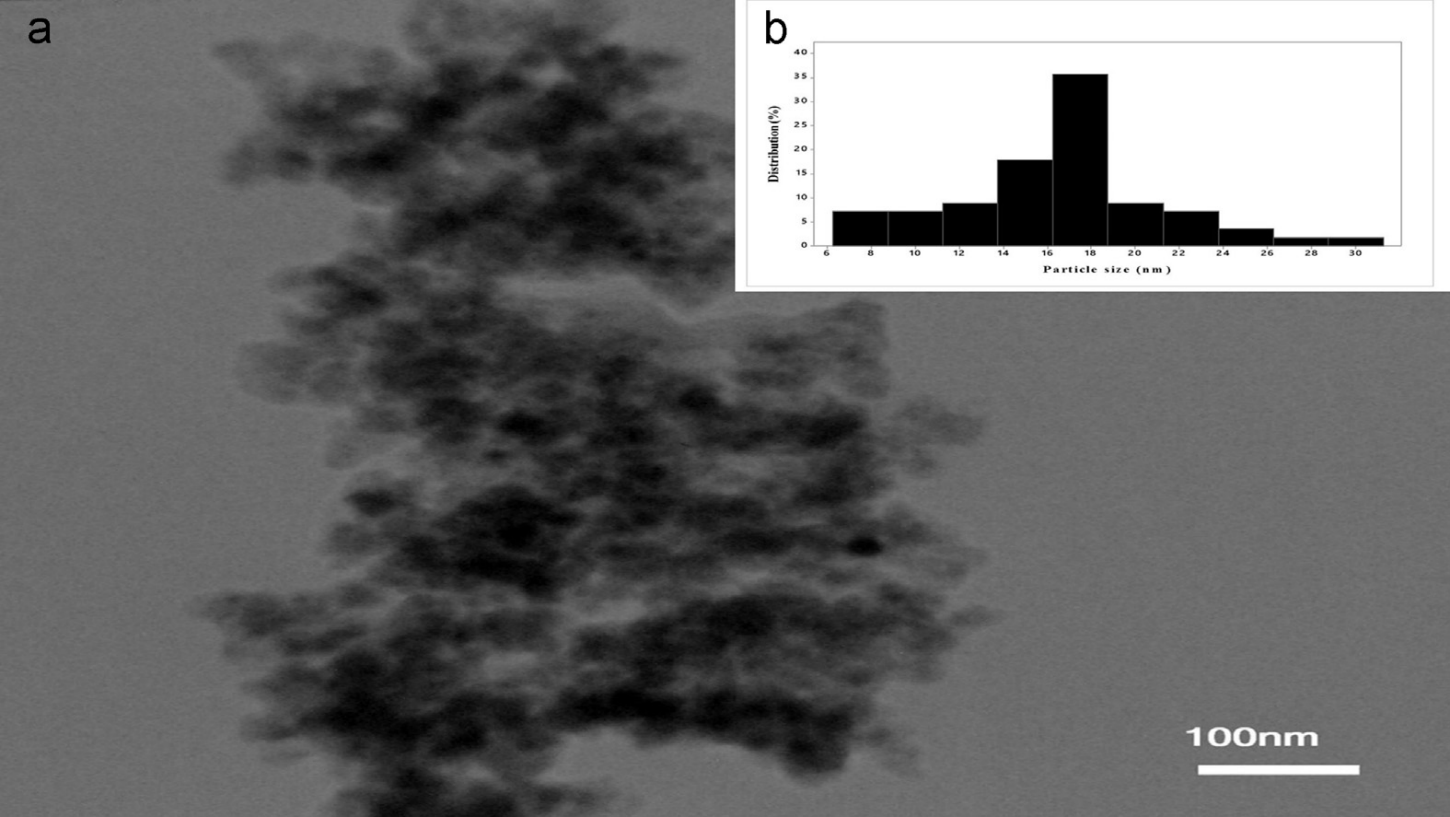
(a) TEM image of tyrosinase-MNPs and, (b) the corresponding particle size histogram.

**Fig. 4:**
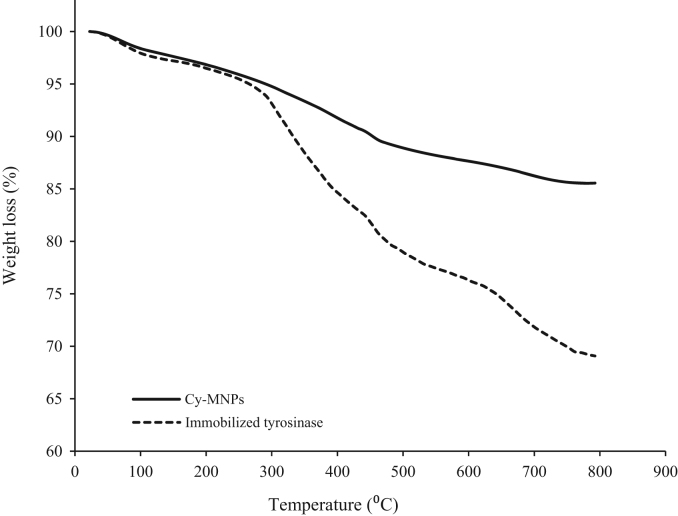
TGA curves of (a) cyanuric chloride functionalized MNPs and, (b) Immobilized tyrosinase.

**Fig. 5:**
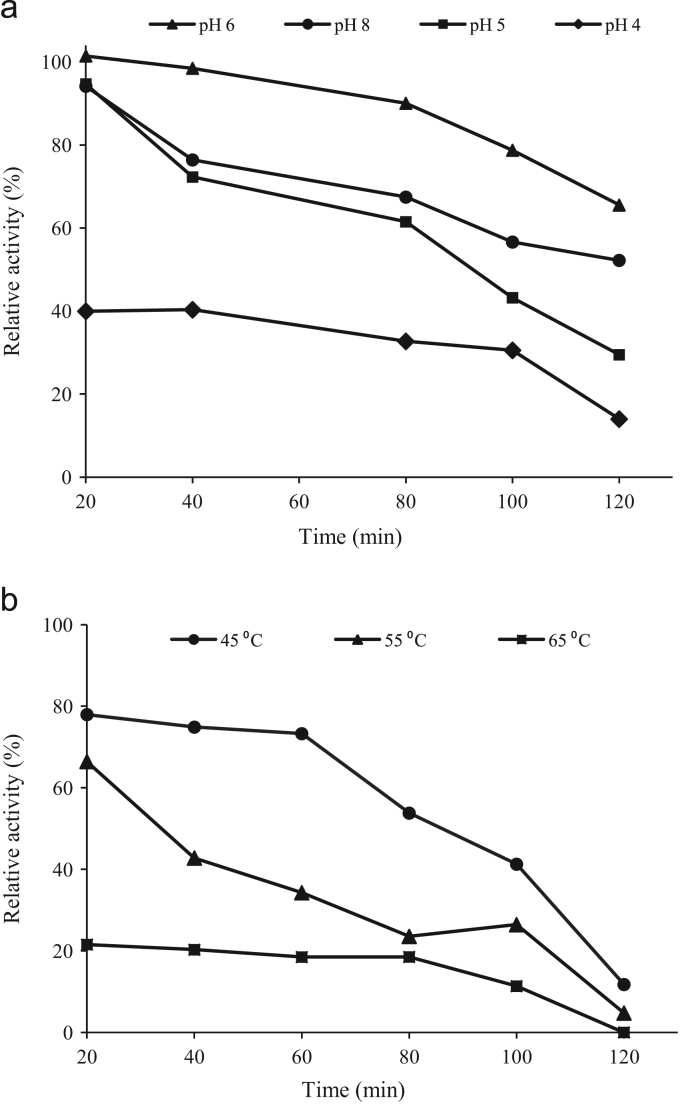
Residual activity of the immobilized tyrosinase after incubation for 120 min at different (a) pH values and, (b) temperatures.

This dataset includes some information regarding purification of extracted tyrosinase from commercial mushroom (*Agaricus bisporus*) such as fold factor in harmony with the applied extraction method (Table 1). The EDX spectra of tyrosinase-MNPs and also the presence of different elements including copper are shown in Fig. 1. The phase purity and crystal structure of synthesized bare magnetic nanoparticles were identified by XRD analysis (Fig. 2). In addition, the morphology of tyrosinase-MNPs and also their average size after immobilization were determined by TEM images and the results were shown in Fig. 3a and b. The weight loss of cyanuric chloride crosslinked magnetic nanoparticles and tyrosinase-MNPs were illustrated in Fig. 4. The activity loss of immobilized tyrosinase after incubation at different pH values and temperatures are represented in Fig. 5a and b.

## 2 Experimental design, materials and methods

### 2.1 Materials

For tyrosinase extraction, the common button mushroom (*Agaricus bisporus*) was purchased from local market. l-DOPA was obtained from Sigma-Aldrich. coomassie brilliant blue G-250, Ferric chloride hexahydrate (FeCl_3_·6H_2_O), ammonium sulfate, ferrous chloride tetrahydrate (FeCl_2_·4H_2_O), cyanuric chloride (Cy), l-tyrosinase, ethanol (99.9%), bovine serum albumin (BSA), ammonium hydroxide solution 25%, tetraethyl orthosilicate (TEOS), 3-Aminopropyltriethoxysilane (APTES) and tetrahydrofuran (THF) were purchased from Merck. Other chemicals were analytical grade. Extraction of tyrosinase from fresh mushroom, synthesizing and surface modification of magnetic nanoparticles and immobilization were carried out as reported [1].

### 2.2 Characterization of nanoparticles

#### 2.2.1 EDX spectra analysis of tyrosinase-MNPs

Immobilization of tyrosinase onto functionalized magnetic nanoparticles was performed according to the literature [1]. A proper amount of immobilized tyrosinase was collected anddried under vacuum at 45 °C for EDX analysis using a Scanning Electron Microscope (SEM) equipped with an EDX detector (TESCAN Vega Model) and the corresponding spectra were presented in Fig. 1.

#### 2.2.2 XRD analysis of bare magnetic nanoparticles

A sample of synthesized bare magnetic nanoparticles was taken and dried under vacuum at 45 °C. Then, the as prepared sample was used for XRD analysis using Bruker D8 Advance, with Cu Kα radiation, *λ*=0.154060 nm instrument with scanning range from 4° to 70° and data was collected at room temperature (Fig. 2).

#### 2.2.3 TEM images of immobilized tyrosinase

In order to highlight the morphology and size distribution of immobilized tyrosinase, about 20 mg oftyrosinase-MNPs were suspended in ethanol solution and then were analyzed by transmission electron microscopy (TEM). Successful silica coating of magnetic nanoparticles, semi-spherical shape and the average size of immobilized tyrosinase were illustrated in Fig. 3a and b.

#### 2.2.4 TGA analysis

Thermo-gravimetric analyses (TGA) were performed by using Netzsch – TGA 209F1 instrument. About 20 mg of cyanuric chloride crosslinked magnetic nanoparticles and tyrosinase-MNPs were used for this analysis. The run was carried out with a uniform heating rate of 10 °C/min from 200 °C to 800 °C under a high purity nitrogen flow (Fig. 4) and the weight loss of the samples was recorded at certain time intervals. Then, the weight loss of samples was plotted as function of temperature which illustrates the differences between these two samples.

### 2.3 Characterization of immobilized tyrosinase

#### 2.3.1 Activity and characterization of extracted tyrosinase

During the extraction procedure, samples were taken from crude extracted solution (first step) and dissolved final precipitate in buffer solution. Then enzyme activity was measured using l-tyrosinase as substrate and also protein content was determined based on a Bradford׳s method [2], [3]. The fold purification and yield of tyrosinase extraction were calculated according to the measured values which were presented in Table 1.

#### 2.3.2 Operational stability of tyrosinase-MNPs

An Appropriate amount of MNPs were added to phosphate buffer solution at different pH values (4.0–8.0) and incubated for 2 h at room temperature to determine the pH stability of the immobilized tyrosinase. Samples were taken in different time intervals and their activities were measured atoptimum condition spectrophotometrically at 475 nm (Fig. 5a). Also, the temperature stability of tyrosinase-MNPs was determined by incubation of the immobilized enzyme in phosphate buffer at different temperature ranging from 25 °C to 65 °C and pH 7.0 and similarly, their residual activities were measured. These results are shown in Fig. 5b.

## Figures and Tables

**Table 1:** Purification of extracted tyrosinase form commercial mushroom *Agaricus bisporus*.

Purification step	Volume (mL)	Total protein (mg)	Activity (U/mL)	Total activity (U)	Specific activity (U/mg)	Fold purification	Yield (%)
Crude	112	82	1205.9	135,060.8	1647.1	1	100
Ammonium sulfate precipitation	10	44	12,316.7	123,167.7	2799.2	1.7	91.2
