# Supplementary material for: Data in support of covalent attachment of tyrosinase onto cyanuric chloride crosslinked magnetic nanoparticles
Source: Data Brief. 2016 Nov 18;9:1098–104. doi: 10.1016/j.dib.2016.11.035 (PMC5128021; doi:10.1016/j.dib.2016.11.035)
Supplement: Supplementary file 1 — Supplementary material [file mmc1.docx]

**Conflict of interest**

The authors declare that there is no conflict of interest.
