# Supplementary material for: Data in support of covalent attachment of tyrosinase onto cyanuric chloride crosslinked magnetic nanoparticles
Source: Data Brief. 2016 Nov 18;9:1098–104. doi: 10.1016/j.dib.2016.11.035 (PMC5128021; doi:10.1016/j.dib.2016.11.035)
Supplement: Supplementary file 2 — Supplementary material [file mmc2.zip › Data in Brief/Table 1.docx]

| Purification step | Volume (mL) | Total protein (mg) | Activity (U/mL) | Total activity (U) | Specific activity (U/mg) | Fold purification | Yield (%) |
| --- | --- | --- | --- | --- | --- | --- | --- |
| Crude | 112 | 82 | 1205.9 | 135060.8 | 1647.1 | 1 | 100 |
| Ammonium sulfate precipitation | 10 | 44 | 12316.7 | 123167.7 | 2799.2 | 1.7 | 91.2 |

**Table 1.** Purification of extracted tyrosinase form commercial mushroom *Agaricus bisporus*.
