# Supplementary figures and images for: Data in support of covalent attachment of tyrosinase onto cyanuric chloride crosslinked magnetic nanoparticles
Source: Data Brief. 2016 Nov 18;9:1098–104. doi: 10.1016/j.dib.2016.11.035 (PMC5128021; doi:10.1016/j.dib.2016.11.035)

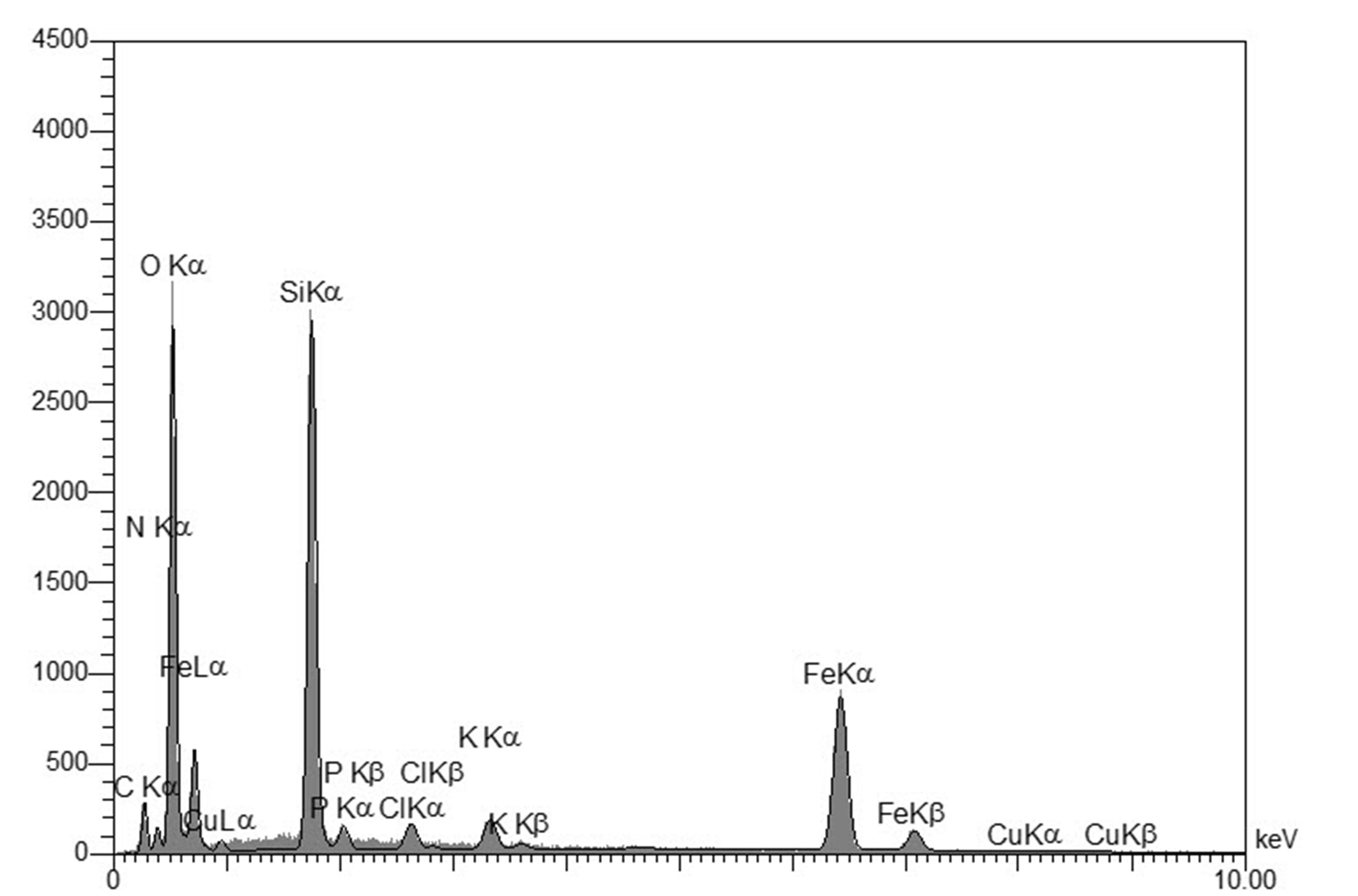

Supplement: Supplementary file 2 — Supplementary material [file mmc2.zip › Data in Brief/Fig. 1.jpg]

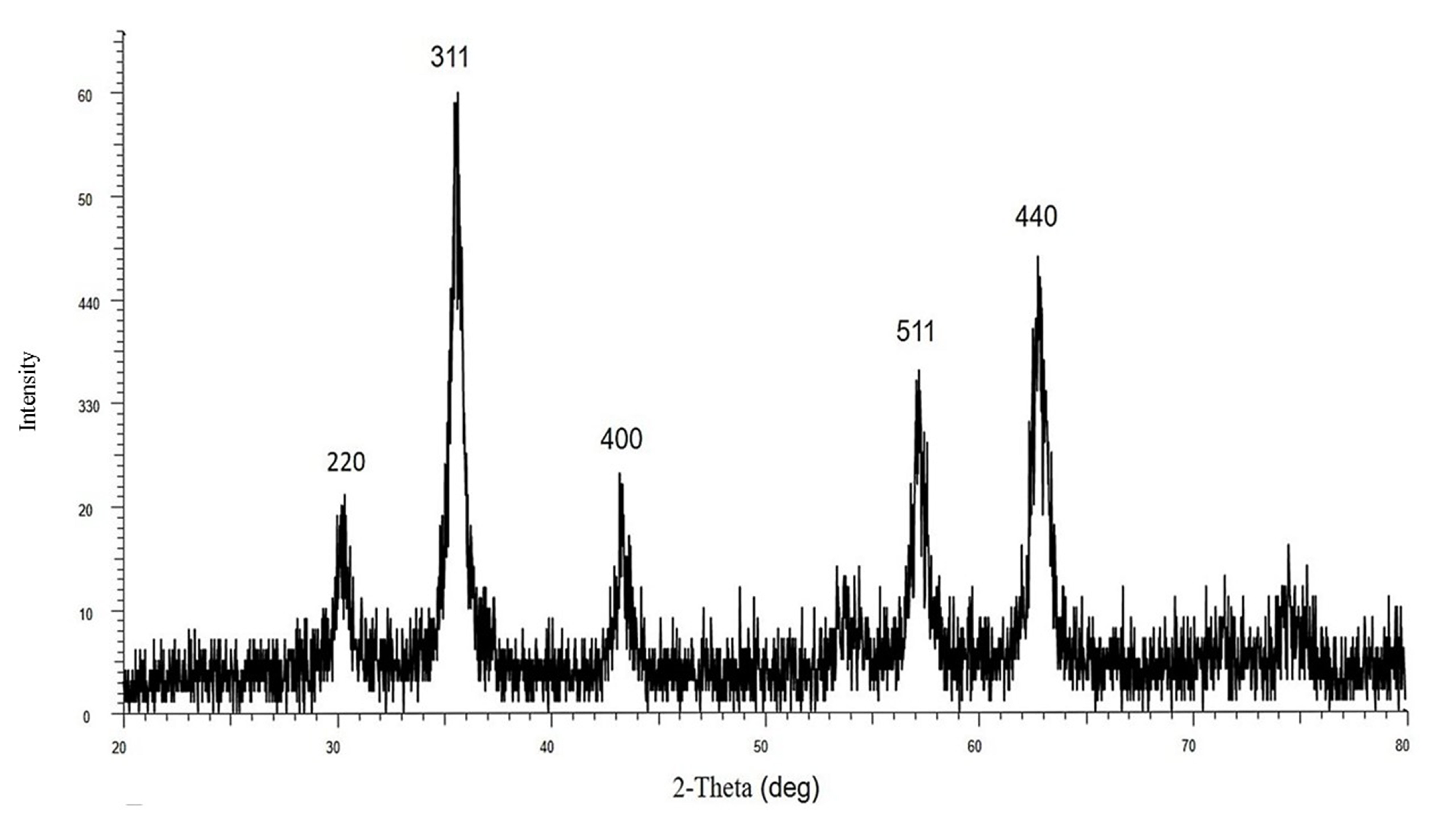

Supplement: Supplementary file 2 — Supplementary material [file mmc2.zip › Data in Brief/Fig. 2.jpg]

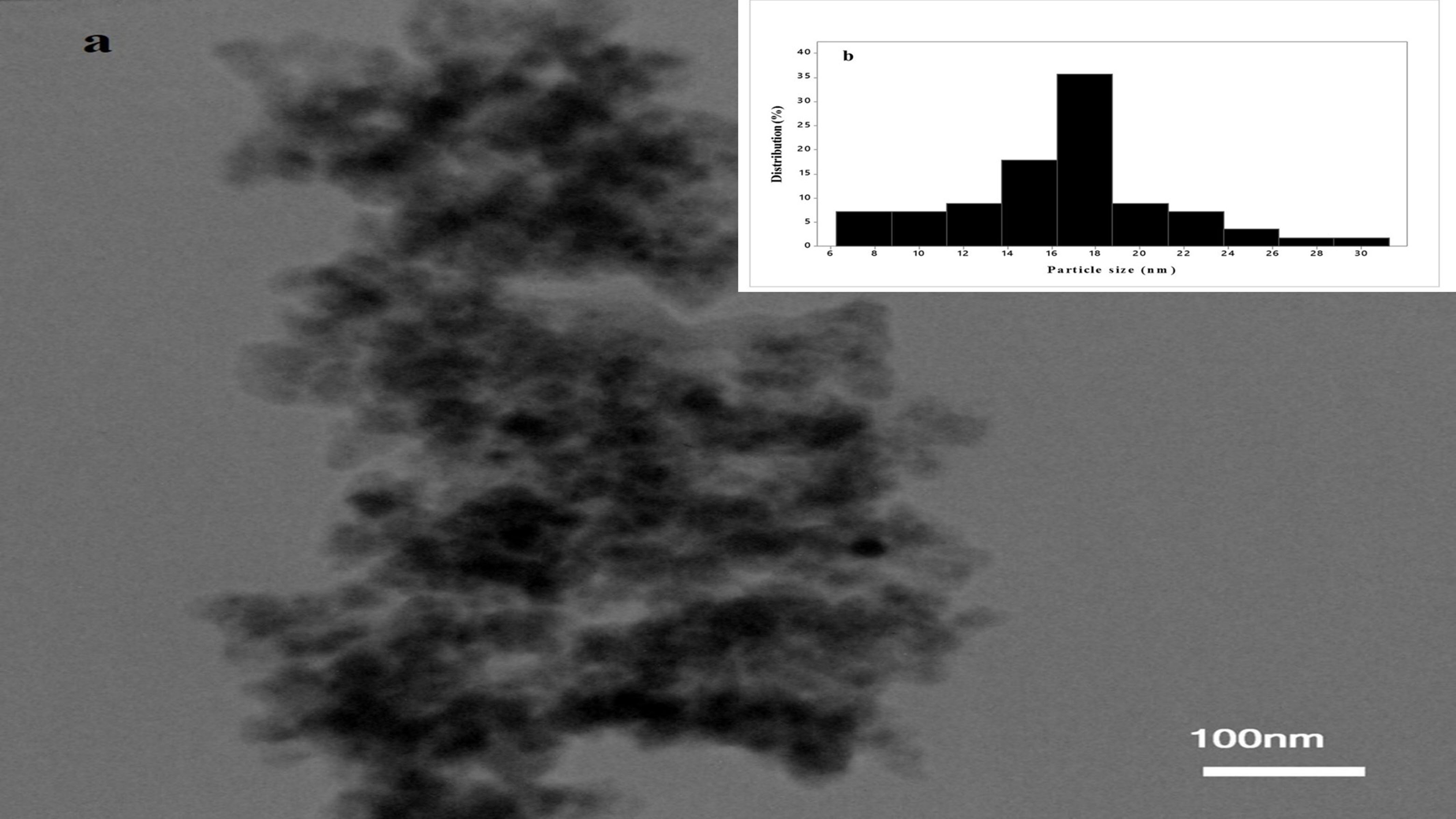

Supplement: Supplementary file 2 — Supplementary material [file mmc2.zip › Data in Brief/Fig. 3.jpg]
